# Temporal Changes in the Faecal Microbiota of Beef Cattle on Feedlot Placement

**DOI:** 10.3390/ani12192500

**Published:** 2022-09-20

**Authors:** Brianna N. Maslen, Lesley A. Gray, Seyed A. Ghorashi, Jason D. White, Michael A. Campbell, Sameer D. Pant

**Affiliations:** 1 Graham Centre for Agricultural Innovation, NSW DPI and Charles Sturt University, Wagga Wagga, NSW 2650, Australia; 2 School of Agricultural, Environmental and Veterinary Sciences, Charles Sturt University, Wagga Wagga, NSW 2650, Australia; 3 Australian Genome Research Facility, Melbourne, VIC 3000, Australia; 4 Office of the Pro Vice Chancellor Research and Innovation, Charles Sturt University, Wagga Wagga, NSW 2678, Australia

**Keywords:** beef cattle, dietary change, faecal, feedlot induction, microbiome, gut microbiota, angus, stress

## Abstract

**Simple Summary:**

Microorganisms inhabiting the gut influence the health and productivity of livestock. Studies in ruminants including beef cattle show that ruminal microorganisms can help convert poor-quality or indigestible feed into nutrients, thereby influencing economically important traits like feed conversion efficiency and meat quality (e.g., marbling), and also interact with receptors on intestinal epithelial cells to regulate a variety of physiological processes including immune responses. Studies have also shown that the proportions of different groups of microorganisms in the gut are influenced by exposure to stress. In beef cattle, given that most cattle are finished on feedlots, induction into feedlots can expose cattle to stress due to transportation, change in diet and change in the handling and management of animals. Therefore, the primary objective of this study was to characterise how gut microorganisms change over time when cattle are inducted into feedlots. The results indicate that there are significant changes in the profiles of gut microorganisms in cattle that are visible soon after feedlot placement. These changes primarily include a reduced diversity of microorganisms within the gut of individual cattle and increased differences in the types of microorganisms that inhabit the gut of different cattle.

**Abstract:**

The microbial communities that inhabit the intestinal tract play an important role in modulating health and productivity. Environmental stressors can impact microbial communities, which can significantly influence host physiology. Cattle are subjected to several environmental stressors when placed on feedlots, such as transportation stress, exposure to feedlot environments and change in diet and management. Exposure to these stressors could influence host gut microbiota, which in turn, could potentially influence host health and performance. The aim of the current study was to characterise the temporal changes that occur in intestinal microbiota as a consequence of feedlot placement by profiling 16s rRNA sequences in rectal faecal samples. When faecal microbiome profiles were compared in terms of relative abundances and alpha diversity metrics, the results showed significant, observable changes in profiles 2 days post-feedlot induction. Furthermore, beta-diversity analysis indicated that the phylogenetic similarity between samples significantly decreased on day 2 (PERMANOVA, *p* < 0.001). These trends were suggestive of a short-term reduction in microbial diversity coupled with decreased similarity between animals. These changes warrant further investigation and could provide opportunities for improved performance, health and even welfare of feedlot cattle in future.

## 1. Introduction

Diverse communities of microorganisms consisting of bacteria, fungi, archaea, viruses and protozoa inhabit different parts of the bodies of animals, including livestock [1]. These microbial communities, commonly referred to as microbiota, are known to modulate a broad range of host physiologies including digestion and metabolism, the regulation of immune responses, the maintenance of epithelial health and energy homeostasis [1,2,3,4]. Consequently, changes in the type and abundance of different groups of microorganisms can significantly impact host physiology, which in the context of livestock can influence performance and productivity [5]. 

The microorganisms inhabiting the gut are particularly relevant for host health and performance. Studies performed using germ-free mice that lack gut microbiota have shown that these mice have impaired immune responsiveness [6,7,8] and reduced weight gain [9]. Furthermore, it has been shown that when the gut microbiota from conventional mice is used to colonise the gut of germ-free mice, it results in a significant increase in body fat and weight, which suggests that gut microbiota contribute to the harvesting of energy [9].

Specifically in livestock, the gut microbiota have been shown to influence economically important traits that relate to health and production. For example, studies have shown that milk yield and composition are significantly correlated to the type and abundance of rumen microbiota in dairy cattle [10]. In beef cattle, rumen microbiota have been found to be significantly associated with marbling, with higher species richness correlated to higher marbling scores [11]. 

In addition to ruminal microflora, there are also a large number of microorganisms that inhabit the intestines and hindgut. These microorganisms also modulate a range of physiological processes that are important for livestock production and health [12]. For example, a recent study in beef cattle showed that hind gut microbiota influences both growth and immune responsiveness [13]. The same study demonstrated that the type and abundance of different groups of microorganisms in the hind gut may be a heritable trait and that genetic selection for desirable profiles could be possible. 

Microbial populations in the gut interact with intestinal epithelia, triggering both innate and adaptive immune responses that keep microbial populations in check [8]. Under normal conditions, this bi-directional relationship is homeostatic, and crucial in maintaining host health and performance, and if this homeostasis is disrupted, dysbiosis can occur. Dysbiosis is a term generally associated with imbalances in microbial compositions, changes in microbial metabolites or changes in microbial distribution within the gut [14,15]. Dysbiosis can frequently provide opportunities for pathogenic microorganisms to proliferate, which can impair host health and performance. For example, several murine studies have reported links between innate immune deficiency and dysbiosis associated with intestinal microbiota [8,16]. 

Changes in the type and abundance of gut microbiota have also been observed after periods of stress, dietary changes, antibiotic usage, disease and altered GIT peristalsis [17,18,19,20]. Regarding beef cattle, they are often finished in feedlots for optimal growth and production before slaughter. However, feedlot placement also represents a period of stress for cattle, as they are subjected to transport-associated stress as well as changes in diet and management, and they are exposed to new environments and cattle. Exposure to these stressors likely contributes to production losses and could also underlie the disease susceptibility that is known to occur within the first four weeks of feedlot placement [21]. However, the mechanisms that mediate the effect of these stressors on animal health and production are not yet well understood. Given that dietary changes and exposure to stress are both known to influence gut microbiota, it is possible that gut microbiota mediate, at least in part, the negative effects of feedlot placement on animal health and production. It is therefore hypothesised that there is a temporal change in gut microbiota of cattle during feedlot transition that can provide novel insights into the physiology of gut-microbiota interactions. The objective of this study was to characterise temporal changes that occur in hind-gut microbiota as a consequence of feedlot placement by profiling 16s rRNA sequences in rectal faecal samples.

## 2. Materials and Methods

This experiment was conducted at Teys feedlot in Jindalee, New South Wales (NSW), Australia. The experimental protocols were reviewed and approved by Charles Sturt University’s Animal Care and Ethics Committee (reference number: A18037).

### 2.1. Animal Husbandry, Experimental Design and Sampling of Cattle

A total of 30 Angus cattle, between 15 to 18 months of age that were transported together ~200 kms from a single commercial herd in Mt Stromlo (ACT, Australia) to Teys feedlot at Jindalee (NSW Australia), were used for sampling. All 30 cattle were inducted via routine procedures (vaccinated and ear tagged) the day after arrival to the feedlot and rectal faecal samples were collected during induction for the first time (day 0). Subsequently, the same animals were sampled again on days 2, 7 and 14 post induction. All thirty animals used for sampling were housed in a separate pen for the two-week period of sampling. While sampled animals were housed separately, their management and feeding was identical to all other animals at the feedlot. Animals had access to oaten hay on arrival to the feedlot for the first night prior to induction and were then offered a ration containing: 42% steam flaked barley, 8% white cottonseed, 5% straw, 17.5% Lucerne hay, 4.5% starter supplement (molasses based), 18% distillers syrup and 5% almond hulls, for the duration of the sampling period. Samples were stored in dry ice immediately after collection and transported to the laboratory where they were stored at −20 °C until further processing. On the final day of sampling (day 14), lung auscultation scores were determined by the feedlot staff on a 5-point scale using a stethoscope to determine animals with early signs of lung pathology [22]. Fifteen cattle that did not demonstrate any lung pathology and had similar faecal consistencies were selected for deoxyribonucleic acid (DNA) extraction from rectal faecal samples and subsequent 16s rRNA sequencing.

### 2.2. DNA Extractions of Faecal Samples and 16s rRNA Sequencing 

Genomic microbial DNA was extracted from faecal samples using the Quick-DNA™ Fecal/Soil Microbe Miniprep Kit (Zymo Research, Irvine, CA, USA) as per manufacturer’s instructions. Final yield and quality of extracted DNA were determined via a NanoDrop 2000 Spectrophotometer (Thermo Scientific, Victoria, VIC, Australia), and subsequently, samples were subjected to paired-end 16s rRNA sequencing at the Australian Genomics Research Facility (AGRF, Queensland, QLD, Australia) on an Illumina MiSeq platform using the primer set of 341F (CCTAYGGGRBGCASCAG)-806R (GGACTACNNGGGTATCTAAT). Of the total 60 samples (15 cattle sampled on day 0, 2, 7 and 14), 58 samples were determined to pass quality control at the AGRF sequencing facility. The remaining 14 2-day samples did not pass quality control and were not subjected to 16s rRNA sequencing. 

### 2.3. 16s rRNA Sequencing and Community Analysis

Paired-ends reads were assembled by aligning the forward and reverse reads using PEAR [23] (version 0.9.5, Heidelberg, Germany). Primers were identified and trimmed. Trimmed sequences were processed using Quantitative Insights into Microbial Ecology (QIIME 1.8, Flagstaff, AZ, USA) [24] USEARCH [25,26] (version 8.0.1623, Tiburon, CA, USA) and UPARSE software [27]. Using USEARCH tools, sequences were quality filtered, and full-length duplicate sequences were removed and sorted by abundance. Singletons or unique reads in the data set were discarded. Sequences were clustered followed by chimera filtering using the “rdp gold” database as reference. To obtain number of reads in each operational taxonomic unit (OTU), reads were mapped back to OTUs with a minimum identity of 97%. Taxonomic classification of OTUs and analysis of alpha diversity metrics were both carried out in QIIME v1.8. Taxonomic classification was carried out using Greengenes databased [28] (Version 13_8, South San Francisco, CA, USA, August 2013) for reference 16s rRNA gene sequences. OTUs were grouped into different taxonomic levels, i.e., phylum, class, order, family, genus and species levels, and relative abundance data at different taxonomic levels was subsequently imported in R [29] to generate relative abundance plots using the ggplot2 [30] and ggpubr [31]. Rarefaction analysis was undertaken to assess both coverage depth and alpha diversity (diversity within groups i.e., day of sampling) using the alpha_rarefaction.py script in QIIME v1.8 [24]. The observed OTU count per unit of sequencing depth was used to assess the adequacy of coverage depth, and species richness was assessed using the Chao1 index [32,33,34]. Beta diversity (diversity between samples) was estimated using unweighted UniFrac distances between samples, and subsequently visualized using principal coordinates analysis (PCoA) plots generated in EMPeror [35]. Statistical analysis to determine whether the temporal differences were significant was carried out using permutational multivariate analysis of variances (PERMANOVAs) in QIIME via the compare_sequences.py script.

## 3. Results

### Temporal Changes in the Bovine Intestinal Microbiota

**Figure 1:**
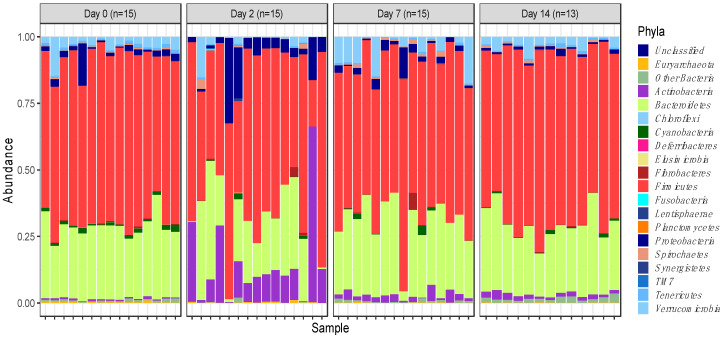
Phylum-level faecal microbiota assortment. Bar chart representing the relative abundance of all bacterial OTUs taxonomically classified at phylum level in each animal sampled on either the day of induction (day 0), or 2-, 7- and 14-days post induction.

A total of 58 samples (n = 15 for days 0, 2 and 7; n = 13 for day 14) were subjected to 16s rRNA sequencing, which yielded a total of 5,959,386 raw sequence reads. These raw reads were subjected to the removal of primer sequences and quality filtering, after which 5,018,090 16s rRNA sequences were retrieved and further analysed. Sequences were clustered in 3,112 OTUs that were assigned to different levels of classification including 19 phyla and 240 genera. However, about 99% of OTUs represented just 5 phyla, i.e., Firmicutes (59.7%), Bacteroidetes (25.2%), Actinobacteria (5%), Verrucomicrobia (3.5%) and Proteobacteria (3.5%) (Figure 1). These can therefore be defined as the ‘core’ phyla making up the microbiome.

**Figure 2:**
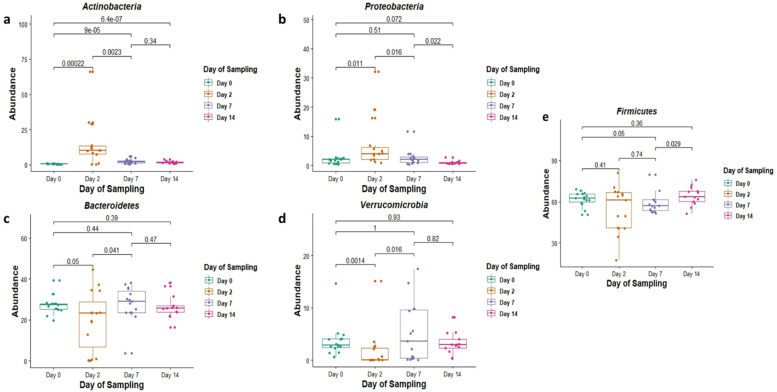
The relative abundance (in %) of the phyla (**a**) *Actinobacteria*, (**b**) *Proteobacteria*, (**c**) *Bacteriodetes*, (**d**) *Verrucomicrobia* and (**e**) *Firmicutes* in faecal DNA on either the day of induction (day 0), or 2, 7 and 14 days post induction (days 2, 7 and 14).

Actinobacteria (5%) and Proteobacteria (3.5%), while having the lowest overall abundance in the core group of phyla, were still found to significantly increase in abundance, particularly on day 2 of feedlot placement (*p* = 0.00022 and *p* = 0.011, respectively) (Figure 2a,b). Increases in abundance of these phyla were observed concurrent to a decrease in the relative abundance of other more abundant phyla.

The overall temporal shifts in the relative abundance of faecal microbiota were best visible by observing the relative abundances of OTUs assigned to different phyla (Figure 1). The relative abundances were found to be significantly altered as soon as 2 days post induction, and while the relative abundances were also altered at days 7 and 14, the magnitude of change was the highest 2 days post induction.

Of these core phyla on day 2, the relative abundances of Bacteroidetes and Verrucomicrobia were significantly lower (*p* = 0.05 and 0.0014, respectively) (Figure 2c,d), and those of Actinobacteria and Proteobacteria were significantly higher (*p* = 0.00022 and 0.011, respectively) when compared with day 0 (Figure 2a,b). The relative abundance of Firmicutes, on the other hand, was not significantly different when compared to day 0 (*p* = 0.41) (Figure 2e).

Similarly, on day 7 when compared with day 0, the relative abundance of phylum Actinobacteria was significantly higher (*p* = 0.0009, and Firmicutes was significantly lower (*p* = 0.05) (Figure 2a,e). The relative abundances of Proteobacteria, Bacteroidetes and Verrucomicrobia did not significantly differ on day 7 when compared with day 0 (*p* = 0.51, 0.44 and 1 respectively) (Figure 2b–d).

Finally, on day 14, the relative abundances of most phyla, including Proteobacteria, Bacteroidetes, Verrucomicrobia and Firmicutes, did not differ significantly compared with day 0 (*p* = 0.072, 0.36, 0.39 and 0.93, respectively) (Figure 2b–e). The only phylum with a significant change in relative abundance when comparing day 14 with day 0 was Actinobacteria (*p* = 0.00000064) (Figure 2a).

**Figure 3:**
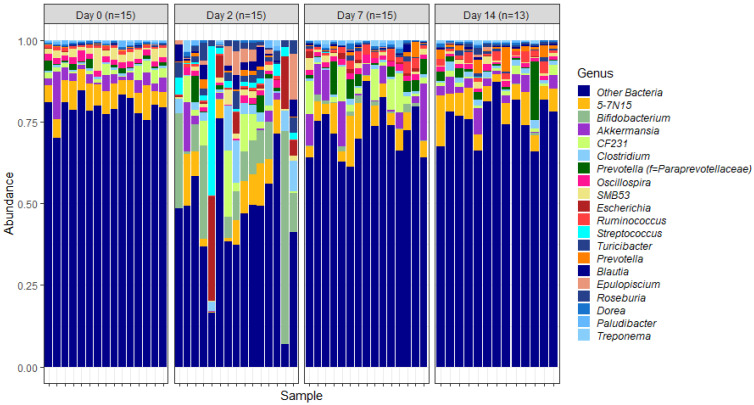
Genus-level faecal microbiota assortment. Bar chart showing the average relative abundance of all bacterial OTUs taxonomically classified at genus level in each animal sampled either on the day of induction (day 0) or 2, 7 and 14 days post induction.

To examine the temporal shifts in faecal microbiomes in depth, the relative abundances of OTUs assigned to different genera were plotted (Figure 3). This indicated similar trends to those observed at the phylum level of classification. The overall trend was suggestive of significant changes in the relative abundance of genera as soon as 2 days post induction.

**Figure 4:**
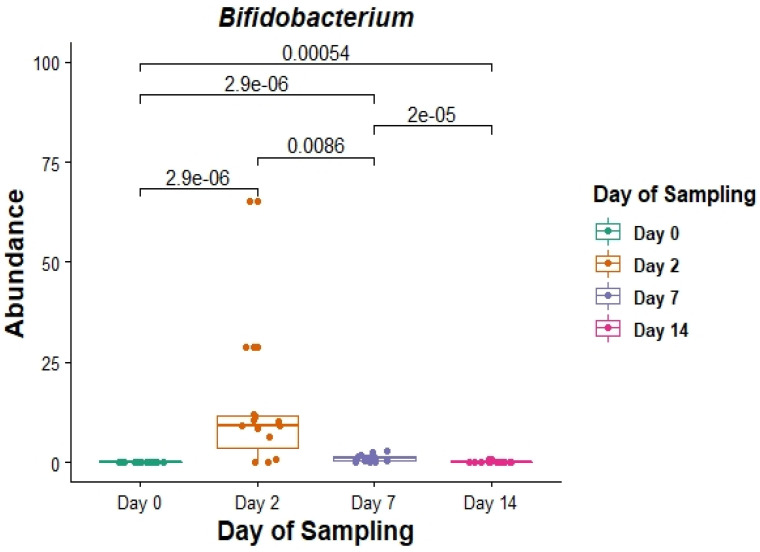
The relative abundance (in%) of the genus *Bifidobacterium* in faecal DNA either on the day of induction (day 0) or 2, 7 and 14 days post induction (days 2, 7 and 14).

Of these core genera (Figure 3), the relative abundance of *Bifidobacterium* was significantly different on days 2, 7 and 14 post induction, when compared with the relative abundance on day 0 (*p* = 0.0000029, 0.0000029 and 0.00054 respectively) (Figure 4).

**Figure 5:**
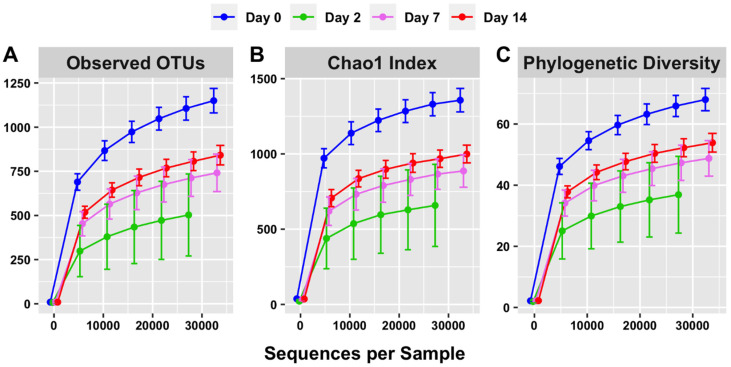
Alpha diversity (within-sample) rarefaction curves measuring species richness of the intestinal microbiota on days 0, 2, 7 and 14. Observed OTUs (**A**), Chao 1 (**B**) and phylogenetic diversity (**C**) relate to the numbers of different microorganisms present in a sample (diversity) compared with how many sequences per sample were used. Error bars are staggered and representative of range.

Rarefaction curves were constructed to evaluate both the depth of sequencing and species richness. When the observed numbers of OTUs were plotted against defined numbers of sequences per sample, the rarefaction curves were relatively shallow at a sequencing depth of 27–33 thousand sequences (Figure 5A). This indicated that further sequencing would result in relatively low gains in species richness and that the depth of sequencing in this study was sufficient to cover a majority of microbial species.

The rarefaction curve representing the Chao1 index (Figure 5B) indicated that species richness was the highest on day 0 of sampling (1325.68) and lowest on day 2 of sampling (665.91) when considering 27,545 sequences. The species richness increased on day 7 of sampling relative to day 2 (868.33) and further increased on day 14 of sampling (968.65). Overall, this is consistent with the trend of short-term dysbiosis, which is also observed in the relative abundance data (Figure 1 and Figure 3). 

The phylogenetic diversity, represented by the third rarefaction curve (Figure 5C), displays a similar trend. The phylogenetic diversity curve indicates a visible difference between days of sampling, with richness observed highest on day 0 and lowest on day 2. Overall, this is consistent with the trends visible in both the OTU (Figure 5A) and Chao 1 (Figure 5B) rarefaction curves. 

**Figure 6:**
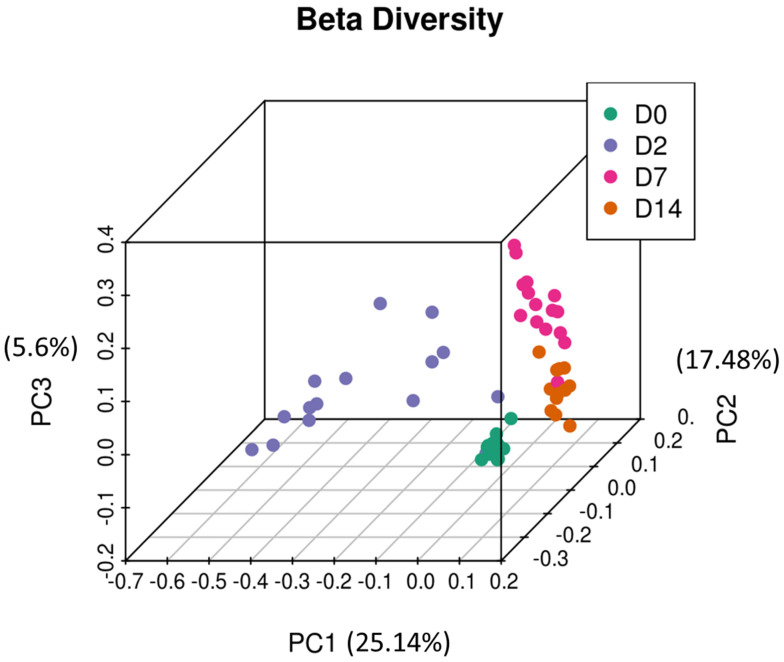
Principal coordinate analysis of the intestinal microbiota of feedlot cattle. Plots of unweighted UniFrac distances by sampling time displays the beta diversity (between-sample phylogenetic similarity) for these time points. Each colour represents a different day that cattle were sampled on, and each dot represents an individual sample. The dots that appear more closely together are more similar. The percent variation explained by the principal coordinates is indicated on the axes.

Principal coordinate analysis (PCoA) was performed to infer beta diversity and between-sample phylogenetic similarity using unweighted UniFrac distances that emphasise the presence or absence of OTUs. The first three principal components, which represented a substantial proportion of the overall variance in the dataset (48.22%), were used to visualise the data (Figure 6). Day 0 samples were found to be tightly clustered, whereas day 2 samples were the least clustered. Day 7 and day 14 samples had intermediate clustering. Overall, this is consistent with trends observed in the relative abundance data and analysis of alpha diversity metrics, broadly indicating a short-term disturbance in intestinal microbiota on day 2 and subsequent recovery on days 7 and 14. PERMANOVA showed that clustering between time points was statistically significant, which indicated structural differences among the four different sampling points (*p* < 0.001).

## 4. Discussion

Microorganisms inhabiting the gut can modulate a broad range of host physiologies that are impacted when the profile (i.e., type and abundance) of gut microorganisms is altered. In livestock, a variety of factors including dietary changes or exposure to stress, can contribute to such alterations in gut microbiota profiles [17,18], which in turn can significantly impact both animal performance (e.g., growth) and health (e.g., immune responsiveness) [13]. Therefore, characterising changes in gut microbiota profiles in livestock in response to a variety of environmental stimuli can provide novel insights and opportunities to improve animal health, performance and welfare. 

In the context of beef cattle, feedlot transition (first 3–4 weeks after arrival of cattle at a feedlot) represents a critical phase in management during which cattle are exposed to a variety of factors (e.g., changes in management/handling or diet) that can impact their growth performance or health. Significant changes in the diet and management of animals during feedlot transition also occur in a relatively short period of time, which can expose cattle to stress. Exposure to stress can impact animal performance and health and can also alter the profile of gut microbiota [17,18,36,37]. Given that both stress and dietary changes can influence the composition of gut microbiota, it is possible that alterations in gut microbiota profiles underlie, at least in part, the impacts of feedlot transition on animal performance and health. Therefore, the objective of this study was to characterise the temporal changes in the faecal microbiota of beef cattle in the first two weeks of feedlot transition. 

In the present study, several alterations in faecal microbiota of cattle were observed within the first two weeks of feedlot placement in terms of relative abundance and alpha and beta diversity metrics. On day 0, the faecal microbiota profiles had relatively high species richness within samples (alpha diversity metrics, Figure 5), coupled with high between-sample phylogenetic similarity (beta diversity, Figure 6), which was apparent after clustering day 0 samples. After induction, on day 2, species richness within samples declined, concurrent with a significant decrease in between-sample phylogenetic similarity (PERMANOVA, *p* < 0.001). Subsequently, on days 7 and 14, species richness within samples increased, and the between-sample phylogenetic similarity gradually increased. Therefore, the overall trend indicated that soon after feedlot placement, the gut microbiota profiles became less diverse and decreasingly similar between individual animals. This short-term trend quickly reversed, and by days 7 and 14 post induction, there was a gradual restoration of gut microbiota diversity; the similarity between gut profiles of individual animals also increased. These short-term changes could have been due to the stress that cattle are exposed to during transportation and induction into feedlots. Previous studies have reported similar findings indicating a decline in species richness as a consequence of exposure to stress [36,37]. Overall, this indicates disruptions in intestinal microbiota homeostasis, and could suggest hind gut dysbiosis [14,15,38].

Commensal bacteria commonly residing in the intestinal tract are predominantly anaerobic members of the phyla Firmicutes and Bacteroidetes, which are not harmful to host health. In fact, the presence of such commensals in the gut limits the opportunity for the proliferation of harmful pathogens, thereby creating a homeostatic balance [14]. In this study, the phylum Bacteroidetes was seen decreasing in abundance 2 days post feedlot induction, which can create opportunities for less common and potentially pathogenic bacteria to proliferate (Figure 4). 

Actinobacteria and Proteobacteria were two key phyla whose abundance was found to increase as a result of feedlot placement (Figure 2a,b). These groups of bacteria are normally found to reside in the gut of different animals, although with relatively low abundances [39]. The phylum Actinobacteria, and associated families like bifidobacteria, are known to contribute to gut barrier homeostasis via the production of short-chain fatty acids (SCFAs) [40]. Proteobacteria, on the other hand, are known to be pathogenic, and the excessive proliferation of these bacteria could negatively impact host health. For example, a relative abundance ratio of Proteobacteria to Firmicutes and Bacteroidetes within the gut microbiota above 0.19 is considered to be suggestive of dysbiosis, which could lead to pathophysiology and impaired immune responses [41,42].

In another study focused on temporal changes in the nasopharyngeal microbiota (NP) on feedlot placement [43], six phyla including Firmicutes, Proteobacteria, Actinobacteria, Bacteroidetes, Tenericutes and Euryarchaeota accounted for 97% of OTU sequences. Results in this current study were similar, with five phyla accounting for 99% of the observed OTU sequences. Tenericutes and Euryarchaeota, found to be core phyla in the NP microbiota, were discovered to have very low abundances in the current study. Similarly, Verrucomicrobia, while not found to be a core phylum of NP microbiota, was discovered to be the fourth most abundant phylum in the faecal microbiota sequenced in this study. These results are consistent with scientific literature, which indicates that Tenericutes and Euryarchaeota are often dominant in the bovine respiratory tract microbiome but have relatively low abundance in the intestinal tract [44,45]. Firmicutes and Proteobacteria, on the other hand, are known to exist as predominant phyla in both the nasopharynx and the intestines [14,43]. 

Assigning OTUs to genera, as expected, showed trends similar to those observed at the phylum level, i.e., significant alterations in faecal microbiota were observed as soon as two days post feedlot placement. This trend is also in agreement with results from the previous study focusing on NP microbiomes. In both studies, *Clostridium*, *Ruminococcus*, *Bifidobacteria* and *Streptococcus* were identified amongst the 20 most abundant genera. Of these, *Bifidobacteria* are of specific interest because of their crucial role in maintaining and promoting gut health. Additionally, previous studies suggest that a reduced abundance of *Bifidobacteria* is indicative of impaired gut health [46,47]. 

In the current study, there was an overall increase in the relative abundance of *Bifidobacteria* two days after feedlot placement, which could indicate that these bacteria proliferate to protect against colonisation by potentially harmful bacteria. However, when comparing relative abundances of individual cattle within the day 2 cohort, it is clear that *Bifidobacteria* do not proliferate in all cattle. Instead, there is significant variability between individual cattle which could, at least in part, contribute to variation in performance or disease susceptibility of cattle during feedlot transition. 

The current study also shows that the direction of change in terms of diversity of microbiota inhabiting different locations of the host can be different [43]. For example, both species richness and within-sample phylogenetic diversity seem to increase in NP microbiomes on feedlot placement, which is contrary to the declines observed in these metrics in faecal microbiomes two days post induction. Feedlots generally expose cattle to dusty environments which are rich in microorganisms. Inhalation of dust and microbes in feedlots could therefore contribute to a short-term increase in alpha diversity, which in turn would explain differences between the two studies. However, there are a few considerations associated with this study that should be taken into account while interpreting results. Firstly, while both this study and a previous study focused on temporal changes in NP microbiota used 4 different time points of sampling (i.e., day 0, 2, 7, 14), the day 0 sampling in this study was conducted on the day of feedlot induction as opposed to the previous study, in which day 0 samples were collected from animals prior to transportation to feedlots. The transportation of animals is known to cause stress, which could also impact the microbial community structures that exist in the intestines [21]. Therefore, it is possible that the day 0 microbiota profiles in this study, derived from faecal samples collected at induction, may represent the influence of stress to some degree. However, it is noteworthy that faecal microbiota profiles on day 0 are still relatively similar across the sampling cohort, compared with day 2 profiles. Furthermore, given that all animals in the sampling cohort came from a single farm, and were exposed to the same stressors (transportation, induction handling, change in diet), the significant increase in the heterogeneity of the faecal microbiota profiles on day 2 suggests significant variability in how hind gut microbiota responds to exposure to stress. Since past studies have already demonstrated that hind gut microbiota is associated with bovine growth and immunity [13], it is plausible that differences in hind gut microbiota profiles underlie at least in part the variability observed in performance and disease susceptibility in beef cattle inducted into feedlots. This warrants further investigation in the future. 

The analysis of beta diversity also indicated a short-term decrease in between-sample phylogenetic similarity two days post induction, which is in agreement with other trends observed in this study (Figure 6). The PCoA indicated that the first three principal components explained a far greater proportion of variance (48.22%) than that observed in the study focused on NP microbiomes of feedlot cattle (23.63%). Previous studies have also reported faecal microbiota to be less sensitive to antibiotic treatment than the NP microbiota, which indicates that profiling faecal microbiota could be used as a complementary tool to investigate the effect of feedlot placement [48].

Gut physiology is generally conserved across species and therefore, it is possible that the mechanisms underlying host-gut microbiota interactions are also conserved. This means that results observed in our study could have broader relevance to other species, including monogastric species.

## 5. Conclusions

Overall, the results from both alpha and beta diversity analysis indicate that there is a loss of taxa and decreased between-sample phylogenetic similarity observable in faecal microbiomes two days post feedlot placement. These declines in the number of taxa could be further investigated because species richness has been previously associated with gut health and stability. A decline in richness could be interpreted as an indicator of a short-term decline in gut health which could have important physiological consequences for the host. Further research is required to investigate whether the variability in temporal changes in faecal microbiomes are associated with health and performance of feedlot cattle. This in turn could afford opportunities to improve performance, health and even welfare.

## Data Availability

The sequencing data is available via NCBI’s Sequence Read Archive (SRA) with the BioProject number PRJNA880985.

## References

[1] Barko P., McMichael M., Swanson K.S., Williams D.A. (2018). The gastrointestinal microbiome: A review. J. Vet. Intern. Med..

[2] Mayer E.A., Tillisch K., Gupta A. (2015). Gut/brain axis and the microbiota. J. Clin. Investig..

[3] Shondelmyer K., Knight R., Sanivarapu A., Ogino S., Vanamala J.K. (2018). Focus: Nutrition and Food Science: Ancient Thali Diet: Gut Microbiota, Immunity, and Health. Yale J. Biol. Med..

[4] Shreiner A.B., Kao J.Y., Young V.B. (2015). The gut microbiome in health and in disease. Curr. Opin. Gastroenterol..

[5] Jones R.M. (2016). Focus: Microbiome: The influence of the gut microbiota on host physiology: In pursuit of mechanisms. Yale J. Biol. Med..

[6] Belkaid Y., Hand T.W. (2014). Role of the microbiota in immunity and inflammation. Cell.

[7] Mazmanian S.K., Liu C.H., Tzianabos A.O., Kasper D.L. (2005). An immunomodulatory molecule of symbiotic bacteria directs maturation of the host immune system. Cell.

[8] Thaiss C.A., Zmora N., Levy M., Elinav E. (2016). The microbiome and innate immunity. Nature.

[9] Turnbaugh P.J., Ley R.E., Mahowald M.A., Magrini V., Mardis E.R., Gordon J.I. (2006). An obesity-associated gut microbiome with increased capacity for energy harvest. Nature.

[10] Jami E., White B.A., Mizrahi I. (2014). Potential role of the bovine rumen microbiome in modulating milk composition and feed efficiency. PLoS ONE.

[11] Kim M., Park T., Jeong J.Y., Baek Y., Lee H.-J. (2020). Association between Rumen Microbiota and Marbling Score in Korean Native Beef Cattle. Animals.

[12] Gopalakrishnan V., Helmink B.A., Spencer C.N., Reuben A., Wargo J.A. (2018). The influence of the gut microbiome on cancer, immunity, and cancer immunotherapy. Cancer Cell.

[13] Fan P., Nelson C.D., Driver J.D., Elzo M.A., Peñagaricano F., Jeong K.C. (2021). Host genetics exerts lifelong effects upon hindgut microbiota and its association with bovine growth and immunity. ISME J..

[14] DeGruttola A.K., Low D., Mizoguchi A., Mizoguchi E. (2016). Current understanding of dysbiosis in disease in human and animal models. Inflamm. Bowel Dis..

[15] Levy M., Kolodziejczyk A.A., Thaiss C.A., Elinav E. (2017). Dysbiosis and the immune system. Nat. Rev. Immunol..

[16] Thaiss C.A., Levy M., Suez J., Elinav E. (2014). The interplay between the innate immune system and the microbiota. Curr. Opin. Immunol..

[17] Bailey M.T., Dowd S.E., Galley J.D., Hufnagle A.R., Allen R.G., Lyte M. (2011). Exposure to a social stressor alters the structure of the intestinal microbiota: Implications for stressor-induced immunomodulation. Brain Behav. Immun..

[18] Karl J.P., Hatch A.M., Arcidiacono S.M., Pearce S.C., Pantoja-Feliciano I.G., Doherty L.A., Soares J.W. (2018). Effects of psychological, environmental and physical stressors on the gut microbiota. Front. Microbiol..

[19] Myers S.P., Hawrelak J. (2004). The causes of intestinal dysbiosis: A review. Altern. Med. Rev..

[20] Walker W. (2017). Dysbiosis. The Microbiota in Gastrointestinal Pathophysiology.

[21] MLA Meat & Livestock Australia—Animal Health. https://www.mla.com.au/research-and-development/feeding-finishing-nutrition/Lotfeeding-intensive-finishing/animal-health/.

[22] Abell K. Evaluation of a Remote Early Disease Identification System to Detect Bovine Respiratory Disease in Beef Cattle in Commercial Australian Feeding Operations. https://www.mla.com.au/research-and-development/reports/2019/evaluation-of-a-remote-early-disease-identification-system-to-detect-bovine-respiratory-disease-in-beef-cattle-in-commercial-australian-feeding-operations--site-1/.

[23] Zhang J., Kobert K., Flouri T., Stamatakis A. (2014). PEAR: A fast and accurate Illumina Paired-End reAd mergeR. Bioinformatics.

[24] Caporaso J.G., Kuczynski J., Stombaugh J., Bittinger K., Bushman F.D., Costello E.K., Fierer N., Peña A.G., Goodrich J.K., Gordon J.I. (2010). QIIME allows analysis of high-throughput community sequencing data. Nat. Methods.

[25] Edgar R.C. (2010). Search and clustering orders of magnitude faster than BLAST. Bioinformatics.

[26] Edgar R.C., Haas B.J., Clemente J.C., Quince C., Knight R. (2011). UCHIME improves sensitivity and speed of chimera detection. Bioinformatics.

[27] Edgar R.C. (2013). UPARSE: Highly accurate OTU sequences from microbial amplicon reads. Nat. Methods.

[28] DeSantis T.Z., Hugenholtz P., Larsen N., Rojas M., Brodie E.L., Keller K., Huber T., Dalevi D., Hu P., Andersen G.L. (2006). Greengenes, a chimera-checked 16S rRNA gene database and workbench compatible with ARB. Appl. Environ. Microbiol..

[29] Team R.C. (2015). R: A Language and Environment for Statistical Computing. 3.2. 2. http://www.r-project.org.

[30] Wickham H. (2016). Data analysis. ggplot2.

[31] Kassambara A. Ggpubr:‘ggplot2’ Based Publication Eady Plots R Package Version 0.2. https://cran.r-project.org/web/packages/ggpubr/index.html.

[32] Chao A. (1984). Nonparametric estimation of the number of classes in a population. Scand. J. Stat..

[33] Chao A., Bunge J. (2002). Estimating the number of species in a stochastic abundance model. Biometrics.

[34] Chao A., Chazdon R.L., Colwell R.K., Shen T.J. (2006). Abundance-based similarity indices and their estimation when there are unseen species in samples. Biometrics.

[35] Vázquez-Baeza Y., Pirrung M., Gonzalez A., Knight R. (2013). EMPeror: A tool for visualizing high-throughput microbial community data. Gigascience.

[36] Wang S.-X., Wu W.-C. (2005). Effects of psychological stress on small intestinal motility and bacteria and mucosa in mice. World J. Gastroenterol. WJG.

[37] Mir R.A., Kleinhenz M.D., Coetzee J.F., Allen H.K., Kudva I.T. (2019). Fecal microbiota changes associated with dehorning and castration stress primarily affects light-weight dairy calves. PLoS ONE.

[38] Tap J., Furet J.P., Bensaada M., Philippe C., Roth H., Rabot S., Lakhdari O., Lombard V., Henrissat B., Corthier G. (2015). Gut microbiota richness promotes its stability upon increased dietary fibre intake in healthy adults. Environ. Microbiol..

[39] Jandhyala S.M., Talukdar R., Subramanyam C., Vuyyuru H., Sasikala M., Reddy D.N. (2015). Role of the normal gut microbiota. World J. Gastroenterol. WJG.

[40] Binda C., Lopetuso L.R., Rizzatti G., Gibiino G., Cennamo V., Gasbarrini A. (2018). Actinobacteria: A relevant minority for the maintenance of gut homeostasis. Dig. Liver Dis..

[41] Auffret M.D., Dewhurst R.J., Duthie C.-A., Rooke J.A., Wallace R.J., Freeman T.C., Stewart R., Watson M., Roehe R. (2017). The rumen microbiome as a reservoir of antimicrobial resistance and pathogenicity genes is directly affected by diet in beef cattle. Microbiome.

[42] Shin N.-R., Whon T.W., Bae J.-W. (2015). Proteobacteria: Microbial signature of dysbiosis in gut microbiota. Trends Biotechnol..

[43] Holman D.B., Timsit E., Amat S., Abbott D.W., Buret A.G., Alexander T.W. (2017). The nasopharyngeal microbiota of beef cattle before and after transport to a feedlot. BMC Microbiol..

[44] Li R.W., Connor E.E., Li C., Baldwin V., Ransom L., Sparks M.E. (2012). Characterization of the rumen microbiota of pre-ruminant calves using metagenomic tools. Environ. Microbiol..

[45] Nicola I., Cerutti F., Grego E., Bertone I., Gianella P., D’Angelo A., Peletto S., Bellino C. (2017). Characterization of the upper and lower respiratory tract microbiota in Piedmontese calves. Microbiome.

[46] Mitsuoka T. (1990). Bifidobacteria and their role in human health. J. Ind. Microbiol..

[47] O’Callaghan A., Van Sinderen D. (2016). Bifidobacteria and their role as members of the human gut microbiota. Front. Microbiol..

[48] Holman D.B., Yang W., Alexander T.W. (2019). Antibiotic treatment in feedlot cattle: A longitudinal study of the effect of oxytetracycline and tulathromycin on the fecal and nasopharyngeal microbiota. Microbiome.

